# Association of benign paroxysmal positional vertigo with meteorological factors in outpatient: a retrospective analysis

**DOI:** 10.3389/fneur.2026.1763908

**Published:** 2026-03-20

**Authors:** Ling Jin, Nannan Si, Kun He, Wei Chang, Jinli Wen, Fengli Cheng, Chang Qing Zhao, Limin Suo

**Affiliations:** 1 Second Hospital of Shanxi Medical University, Taiyuan, China; 2 Heping Hospital Affiliated to Changzhi Medical College, Changzhi, China; 3 Meteorological Disaster Prevention Technology Center of Shanxi Province, Taiyuan, Shanxi, China

**Keywords:** BPPV, daylight duration, humidity, meteorological factor, seasonality

## Abstract

**Background:**

While previous studies have reported associations between benign paroxysmal positional vertigo (BPPV) and meteorological factors, the relationship remains contentious. This study investigates the correlation between key meteorological variables and BPPV incidence through multivariate analysis, providing new insights into its pathogenesis.

**Methods:**

We conducted a retrospective analysis of 1,463 BPPV cases (excluding secondary vertigo) from two tertiary hospitals in Shanxi Province (October 2020–September 2024). Meteorological data (monthly averages of temperature, humidity, atmospheric pressure, precipitation, and sunshine duration) were obtained from provincial meteorological authorities. Normally distributed continuous variables were expressed as mean ± SD and compared using t-tests or ANOVA; non-parametric variables were analyzed with rank-sum tests. Categorical variables were compared via chi-square tests. Multivariate analyses employed polynomial logistic regression (overall cohort) and binary logistic regression (mixed-type BPPV).

**Results:**

The cohort showed female predominance (67.74%), with peak incidence in 51–70-year-olds (51.9%). A statistically significant seasonal variation was observed, with a higher incidence in spring and winter compared to summer and autumn (*p* < 0.05), peaking in March (11.28%) and troughing in October (6.36%). Posterior semicircular canal involvement predominated (68.97%), predominantly right-sided (62.3%). Canalolithiasis accounted for 91.87% versus 4.6% cupulolithiasis. Mixed-type BPPV (*n* = 52) primarily involved posterior+horizontal canal combinations (71.15%) with bilateral predominance (55.77%). Multivariate analysis identified monthly variations (*p* = 0.031) and humidity (χ^2^ = 7.065, *p* = 0.029) as independent predictors of overall BPPV. Mixed-type BPPV demonstrated gender-specific susceptibility (female: OR 2.15, *p* = 0.004), with significant associations to temperature (*β* = −0.31, *p* = 0.018) and daylight duration (OR 1.32, *p* < 0.001).

**Conclusion:**

Meteorological factors, particularly humidity and daylight duration, significantly influence BPPV occurrence with distinct seasonal patterns. The gender-specific susceptibility and thermal/daylight associations in mixed-type BPPV suggest multifactorial pathogenesis. These findings may inform the development of meteorology-aware clinical strategies for BPPV prevention and management, though further research is needed to establish causal links and translate these associations into concrete interventions.

## 1 Introduction

Benign paroxysmal positional vertigo (BPPV), the most common peripheral vestibular disorder, manifests as transient rotational vertigo triggered by specific head position changes. Epidemiological studies underscore its significant prevalence, reporting vestibular vertigo’s lifetime prevalence and incidence at 7.8 and 1.5%, respectively. BPPV specifically exhibits a lifetime prevalence of 2.4%, a 1-year prevalence of 1.6%, and an annual incidence of 0.6% (1). Although non-fatal, BPPV-induced vertigo and imbalance profoundly impact psychological well-being, increasing risks of anxiety, depression, and reduced quality of life (2). Furthermore, it imposes substantial economic burdens on healthcare systems globally, with vertigo-related costs in the US alone reaching an estimated $55 billion in 2020 (3). These factors collectively underscore BPPV’s status as a global public health priority, necessitating a deeper understanding of its clinical characteristics and modifiable risk factors for effective prevention and management strategies.

The accelerating impacts of climate change and intensified human activities have spurred significant interest in understanding the complex interplay between meteorological parameters and human health. A robust body of research confirms associations between weather variables — such as temperature, atmospheric pressure, humidity, and solar radiation — and seasonal rhythms with the incidence and exacerbation of various conditions, including cardiovascular, respiratory, immune, and infectious diseases (4). Investigations into potential correlations between meteorological factors and BPPV incidence have yielded intriguing, yet often contradictory, findings. Several studies report distinct seasonal patterns, frequently observing peak BPPV incidence during colder months (autumn and winter) in both Northern and Southern hemispheres (5). This seasonality is often linked to lower temperatures (6), higher atmospheric pressure (7), and reduced rainfall (8, 9). For instance, studies in Shanghai, China (6), Beijing, China (10), Boston, USA (11, 12), and a southern Brazilian city (9) consistently reported higher BPPV rates in winter/early spring. Conversely, some research, including a study in Kars, Türkiye, observed increased incidence during summer, potentially linked to specific local factors like summer crowding and humidity (7). Notably, other investigations found no statistically significant monthly or seasonal variations (4, 5, 13), highlighting the complexity and potential regional specificity of these relationships.

The observed discrepancies in BPPV-meteorology associations likely stem from methodological limitations in existing research. Key limitations include insufficient multivariate analyses that fail to account for the complex interdependencies among multiple climatic variables simultaneously (13). Many studies rely solely on univariate correlations, which may overlook confounding interactions. Furthermore, variations in study design (retrospective vs. prospective), geographical location (latitude, climate zone), population demographics, diagnostic criteria, and the range of meteorological parameters analyzed contribute significantly to the heterogeneous results (14, 15). Additionally, studies exploring the role of objective neurotological testing in BPPV diagnosis have yielded conflicting findings, further underscoring the complexity of this condition (15). A recent comprehensive meta-analysis involving 16,144 patients (16) attempted to reconcile these findings, revealing a statistically significant positive correlation between BPPV incidence and atmospheric pressure (*p* = 0.003) and a significant negative correlation with rainfall (*p* = 0.017). However, it found no significant overall correlation with temperature, humidity, sunlight time, or solar radiation levels, emphasizing the nuanced nature of these relationships and the need for more sophisticated analytical approaches.

While BPPV is predominantly idiopathic, secondary cases are associated with identifiable factors such as head trauma, Ménière’s disease, and inner ear pathologies. Emerging evidence strongly implicates vitamin D deficiency as a potential modifiable risk factor in BPPV pathogenesis (17). The proposed mechanism involves vitamin D’s crucial role in calcium metabolism. Since otoconia are composed of calcium carbonate crystals, vitamin D deficiency could potentially impair otoconial formation, maintenance, or dissolution, increasing susceptibility to detachment (18). Crucially, serum vitamin D levels exhibit well-documented seasonal fluctuations, largely dependent on sunlight exposure (specifically UVB radiation) necessary for cutaneous synthesis (9). Studies have directly measured lower serum 25-hydroxyvitamin D levels during winter months concurrent with higher BPPV incidence (6), and some have demonstrated a moderate negative correlation between monthly vitamin D levels and BPPV case numbers (6). This provides a plausible biological pathway linking seasonal variations in solar radiation to BPPV occurrence via vitamin D status (6, 8, 9, 12). However, not all studies support this direct link; research in Greece found seasonal variation in BPPV but no significant correlation between BPPV incidence and climatic proxies for vitamin D levels (sunshine hours, solar irradiance) (4, 5). Alternative or complementary explanations for seasonality include reduced physical activity during colder weather potentially influencing otolith stability (8), and the direct biomechanical effects of atmospheric pressure changes on inner ear fluid dynamics or otolith displacement (11, 19). Additionally, emerging factors like air pollution (e.g., carbon monoxide levels) have also shown preliminary associations with increased BPPV risk in specific locales (7), warranting further investigation.

Therefore, significant gaps persist in our understanding of how specific meteorological parameters independently and interactively influence BPPV incidence. The contradictory findings underscore the necessity for studies employing robust multifactorial analyses that simultaneously control for a wider array of climatic variables (e.g., temperature, pressure, humidity, rainfall, sunshine, pollution), key confounders (e.g., age, cardiovascular risk factors (10)), and potential mediators like measured vitamin D levels (6). Our study directly addresses this need by employing both univariate and sophisticated multifactorial approaches to objectively evaluate the relationships between meteorological parameters and BPPV incidence within a defined population and region. By rigorously analyzing these complex interdependencies, we aim to enhance the reliability of our findings and contribute to clarifying the etiological role of climate in BPPV, ultimately informing targeted prevention strategies.

## 2 Materials and methods

### 2.1 Study design and participant selection

Participants were consecutively recruited from the otolaryngology and neurology outpatient clinics, as well as specialized vertigo centers, at two tertiary hospitals in Shanxi Province (October 2020–September 2024). The diagnosis of all BPPV cases was strictly based on the internationally recognized diagnostic criteria of the Bárány Society (18), confirmed by characteristic positioning nystagmus observed during diagnostic maneuvers (e.g., Dix-Hallpike test, Supine Roll Test) and evaluated by experienced specialists. The study adhered to Declaration of Helsinki principles with written informed consent obtained. Diagnosis also referenced the 2017 Chinese BPPV guidelines (19): (1) Recurrent transient vertigo (≤1 min) triggered by gravity-direction changes; (2) Vertigo/nystagmus confirmation via videonystagmography (VNG) positional testing; (3) Exclusion of secondary vertigo. Key exclusion criteria focused on ‘secondary BPPV’, defined as cases with a clear underlying etiology such as head trauma, Ménière’s disease, vestibular neuritis, sudden sensorineural hearing loss with vertigo, inner ear surgery, or other known neurologic disorders affecting the vestibular system. These cases were excluded to focus the analysis on idiopathic BPPV and minimize confounding by known pathophysiological mechanisms. During the study period, the outpatient and vertigo specialty services at both participating centers operated normally without major disruptions or long-term closures due to the COVID-19 pandemic, which we believe minimized systematic bias in patient recruitment. A total of 4,218 patient records with a chief complaint of vertigo or dizziness were initially screened. Among these, 1,572 patients met the clinical diagnostic criteria for BPPV. After excluding 109 patients who met the criteria for secondary BPPV, a final cohort of 1,463 patients with idiopathic BPPV was included in the retrospective analysis.

Meteorological parameters (monthly averages of temperature, humidity, precipitation, atmospheric pressure, and sunshine duration) were obtained from the Shanxi Provincial Meteorological Bureau. These data were collected from standardized weather monitoring stations located within the same geographical region as the participating hospitals, ensuring representative climatic measurements for the study population.

### 2.2 Statistical analysis

SPSS 26.0 (IBM Corp., Armonk, NY, USA) facilitated all statistical analyses. Descriptive statistics included means/SDs for parametric data and frequencies/percentages for categorical variables. The Kolmogorov–Smirnov test was employed to assess the normality of continuous variable distributions. Between-group comparisons utilized: Student’s t-test/ANOVA for normally distributed metrics; Mann–Whitney U/Kruskal-Wallis tests for non-parametric data; Chi-square/Fisher’s exact tests for proportions. For multivariate analysis, multinomial logistic regression (enter method) was employed for the overall cohort, with all meteorological variables (monthly factor, temperature, humidity, pressure, precipitation, sunshine duration) entered simultaneously into the model to assess their independent influence on BPPV incidence. Binary logistic regression was used for the MC-BPPV subgroup analysis. All multivariate models controlled for potential confounding variables, with the significance threshold set at *p* < 0.05 (two-tailed).

## 3 Results

### 3.1 Age characteristics of BPPV onset

A total of 1,463 patients diagnosed with benign paroxysmal positional vertigo (BPPV) were enrolled in this study. Among them, 472 were males (32.26%) and 991 were females (67.74%), with ages ranging from 4 to 91 years. The distribution of cases across different age groups was as follows: <20 years (9 cases), 20–30 years (75 cases), 31–40 years (208 cases), 41–50 years (291 cases), 51–60 years (471 cases), 61–70 years (288 cases), 71–80 years (93 cases), and 81–100 years (28 cases). Notably, 78.13% of the cases occurred in patients aged 41–80 years, while the cohort aged 51–70 years accounted for 51.88% of the total cases.

**Figure 1:**
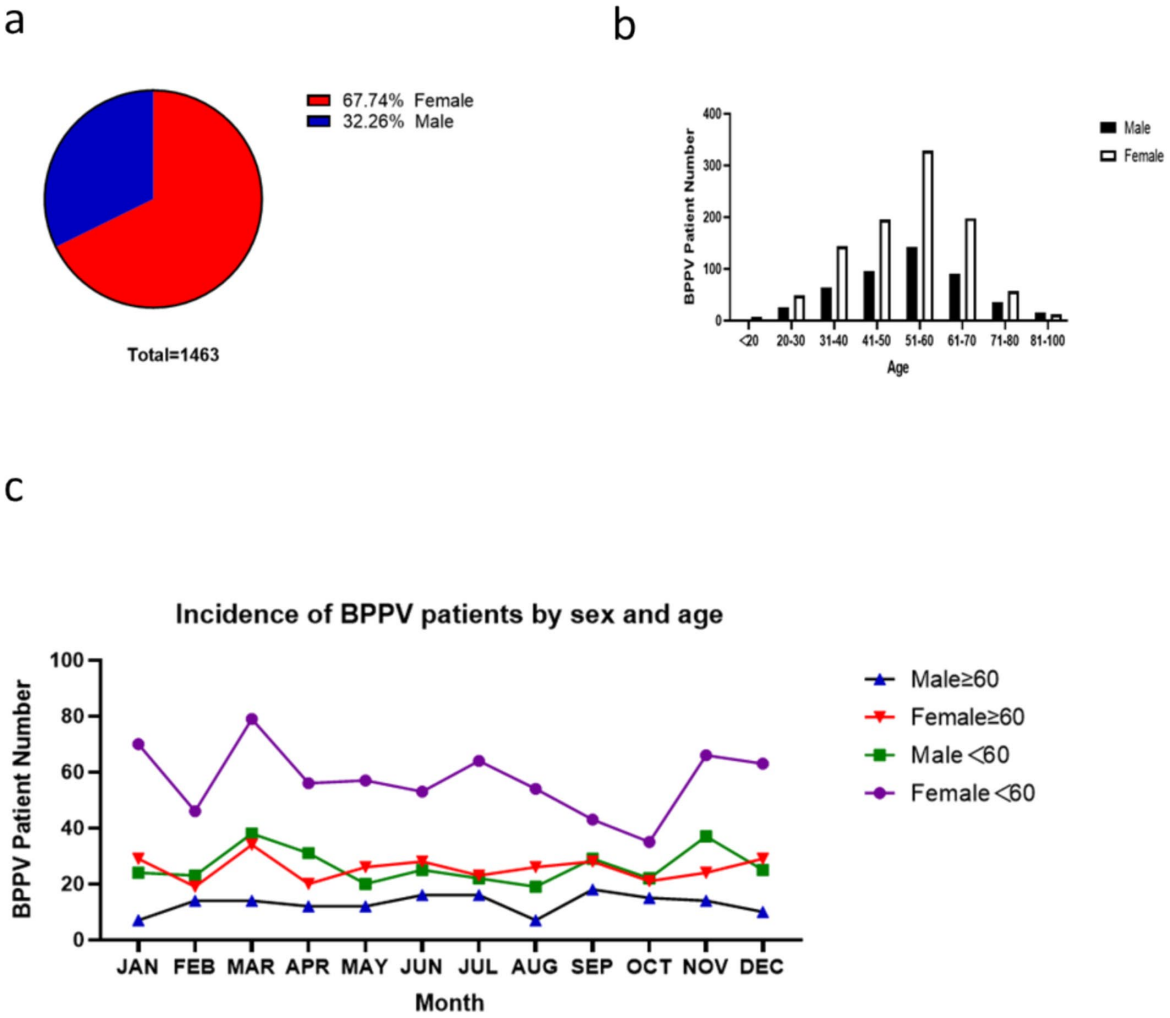
Distribution of BPPV cases by age and gender, and benign paroxysmal positional vertigo is abbreviated as BPPV. The pie chart in panel **(a)** illustrates the gender distribution among the 1,463 patients, with females accounting for 67.74% and males comprising 32.26%. The bar chart in panel **(b)** shows the distribution of BPPV cases across different age groups, with the 51–60 years group having the highest number of cases (471). The chart in panel **(c)** depicts the distribution of BPPV cases stratified by both age group and gender. The findings indicate that females under 60 years constitute the highest-risk demographic.

Analysis of sex-specific trends revealed linear growth in case numbers for both sexes between 20 and 60 years, followed by a marked decline after 70 years. In patients aged over 80 years, no significant sex disparity was observed (Fisher’s exact test, *p* = 0.623). To facilitate comparative analysis, participants were stratified into four categories: females ≥60 years, females <60 years, males ≥60 years, and males <60 years. Key findings indicated that females under 60 years represented the highest-risk demographic, with peak incidence observed in March and trough values in October. Conversely, males aged ≥60 years demonstrated the lowest incidence rates. Similar incidence rates were noted between females ≥60 years and males <60 years (chi-square test, χ^2^ = 2.341, *p* = 0.126). Detailed epidemiological patterns are illustrated in Figure 1.

### 3.2 Characteristics of affected semicircular canals in BPPV

**Figure 2:**
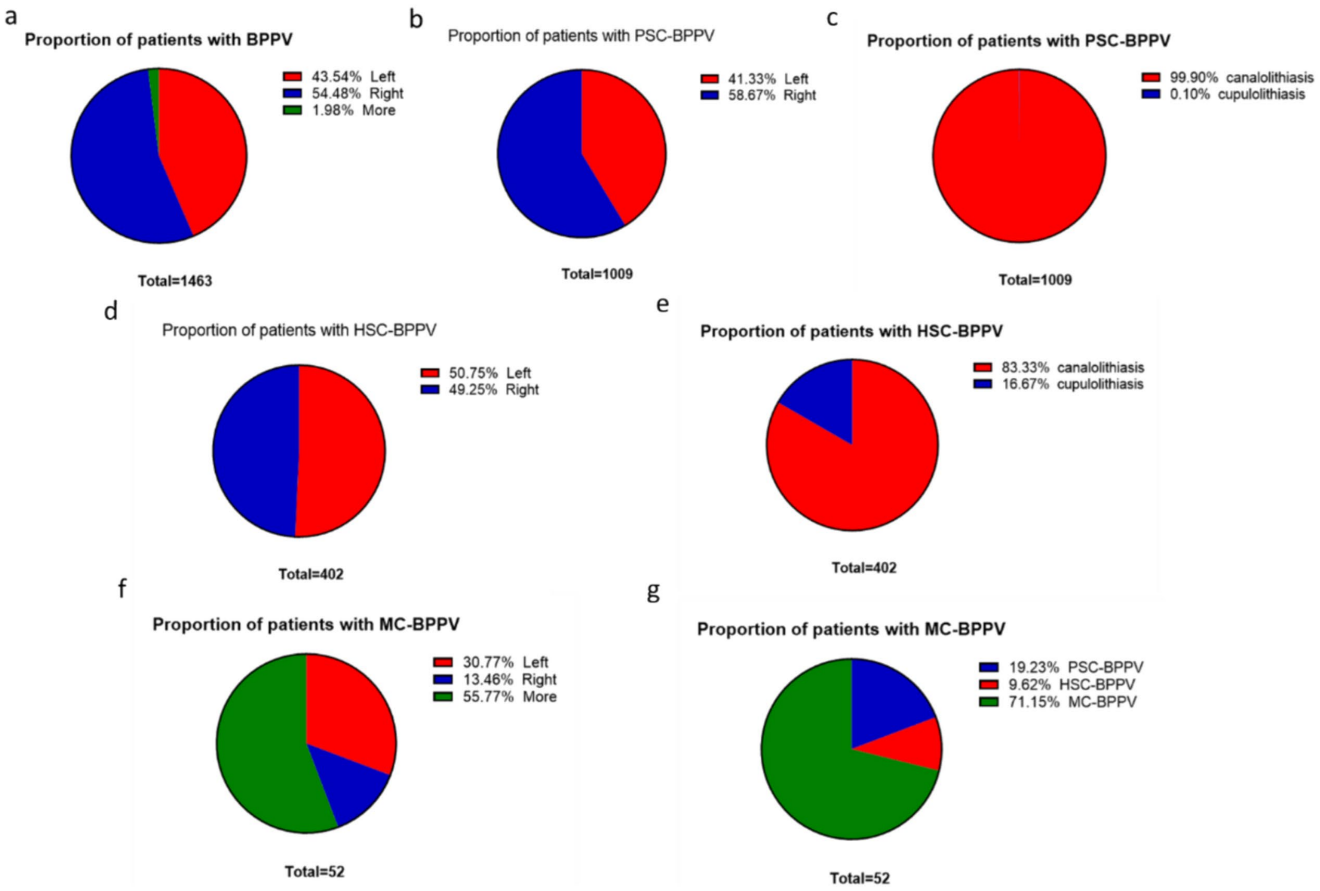
Proportion of patients with BPPV and benign paroxysmal positional vertigo is abbreviated as BPPV. **(a)** Proportion of total patients with BPPV; **(b)** Distribution of PSC-BPPV (posterior semicircular canal BPPV) by side; **(c)** Distribution of PSC-BPPV by type; **(d)** Distribution of HSC-BPPV (horizontal semicircular canal BPPV) by side; **(e)** Distribution of HSC-BPPV by type; **(f)** Distribution of MC-BPPV (mixed-type BPPV) by side; **(g)** Distribution of MC-BPPV by type.

Among the 1,463 BPPV cases analyzed, 797 cases (54.48%) involved the right semicircular canal, 637 cases (43.54%) involved the left semicircular canal, and 29 cases (1.98%) were classified as mixed-type. Canalolithiasis was the predominant form, accounting for 1,344 cases (91.87%), in contrast to cupulolithiasis, which was observed in 67 cases (4.6%). For posterior semicircular canal involvement, there were 417 cases (41.33%) on the left side and 592 cases (58.67%) on the right side. Among these, canalolithiasis was present in 1,008 cases (99.90%), while only 1 case (0.1%) was attributed to cupulolithiasis. In horizontal semicircular canal involvement, 204 cases (50.75%) affected the left side, while 198 cases (49.25%) involved the right side. Canalolithiasis was identified in 335 cases (83.33%), whereas cupulolithiasis accounted for 67 cases (16.67%). Mixed-type BPPV patterns included a variety of combinations: bilateral posterior canals (10 cases, 19.23%), bilateral horizontal canals (5 cases, 9.62%), and posterior-horizontal combinations (37 cases, 71.15%). Among these, left-side dominance was observed in 16 cases (30.77%), right-side involvement in 7 cases (13.46%), and bilateral cases in 29 cases (55.77%) (Figure 2).

### 3.3 Distribution of BPPV onset by month and season

**Figure 3:**
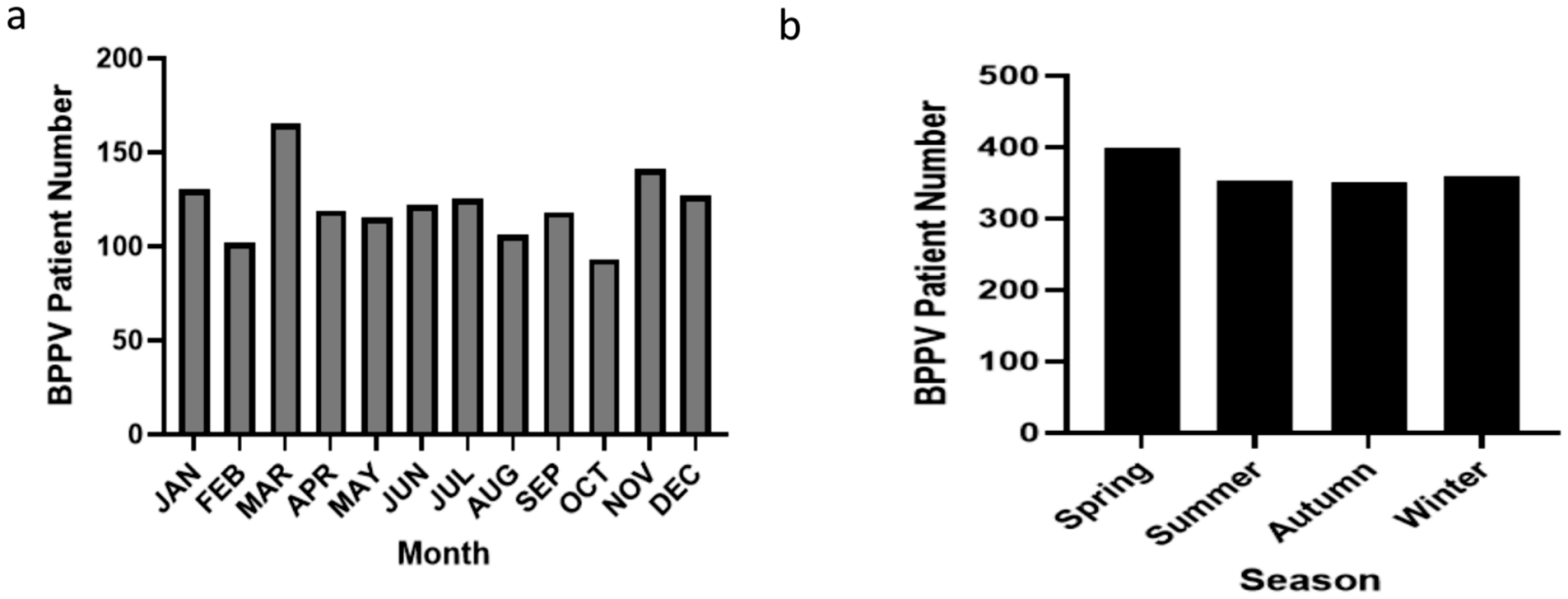
Overall monthly and seasonal distribution of BPPV cases and benign paroxysmal positional vertigo is abbreviated as BPPV. **(a)** Monthly distribution of BPPV cases. The highest incidence was recorded in March (11.28%), while the lowest occurred in October (6.36%); **(b)** Seasonal distribution of BPPV cases. The number of cases was slightly higher in spring and winter compared to summer and autumn.

The monthly distribution of BPPV cases was as follows: January (130), February (102), March (165), April (119), May (115), June (122), July (125), August (106), September (118), October (93), November (141), and December (127). The highest incidence was observed in March, with 165 cases (11.28%), while the lowest occurred in October, with 93 cases (6.36%). Although seasonal variation was not statistically significant, a modest increase in cases was noted during spring (March–May: 399 cases) and winter (December–February: 359 cases) compared to summer (June–August: 353 cases) and autumn (September–November: 352 cases) (chi-square test, χ^2^ = 4.528, *p* = 0.210). These seasonal trends are depicted in Figure 3.

### 3.4 Characteristics of meteorological factors associated with BPPV onset

#### 3.4.1 Univariate analysis with BPPV onset

A total of 1,439 cases were included in the analysis after excluding 24 cases from September and October 2024 due to incomplete meteorological data.

The analysis was conducted across three types of BPPV: posterior and horizontal semicircular canals (multicanal involvement, *n* = 997), horizontal canal alone (*n* = 390), and mixed-type (*n* = 52). The results showed no significant differences in age distribution among the three types, with median ages of 53 years for multicanal involvement, 54 years for horizontal canal, and 53 years for mixed-type (Kruskal-Wallis test, H = 0.107, *p* = 0.948). In terms of sex, there were no significant differences either, with 31.3% males and 68.7% females in the multicanal group, 33.8% males and 66.2% females in the horizontal group, and 26.9% males and 73.1% females in the mixed-type group (chi-square test, χ^2^ = 1.440, *p* = 0.487) (Table 1).

**Table 1:** Univariate analysis with BPPV onset.

Variable	Lateral type			*H*/*χ*^2^ value	*p* value
	PLSC (*n* = 997)	Horizontal (*n* = 390)	Mixed (*n* = 52)		
Age	53 (43, 62)	54 (43, 61)	53 (43, 61)	0.107^a^	0.948
Sex, *n* (%)				1.440^b^	0.487
Male	312 (31.3%)	132 (33.8%)	14 (26.9%)		
Female	685 (68.7%)	258 (66.2%)	38 (73.1%)		
Season				6.649^b^	0.355
Spring	279 (28.0%)	110 (28.2%)	10 (19.2%)		
Summer	244 (24.5%)	91 (23.3%)	18 (34.6%)		
Autumn	232 (23.3%)	82 (21.0%)	14 (26.9%)		
Winter	242 (24.3%)	107 (27.4%)	10 (19.2%)		
Temperature	10.1 (2.8, 20.3)	9.85 (−1.7, 19.25)	13.3 (2.8, 22.2)	3.144^a^	0.208
Humidity	58.9 (51, 72)	58 (50.8, 70)	58.45 (49.15, 74.45)	1.528^a^	0.466
Barometric Pressure	909.1 (903, 922.65)	908.7 (903.2, 923.3)	908.1 (898.7, 920.48)	1.344^a^	0.511
Rainfall	23 (10.4, 66)	23.6 (9,47.3)	23.3 (12.3, 100)	0.586^a^	0.746
Sunshine hours	198.6 (168.4, 220.5)	202.05 (172.5, 219.38)	204.6 (173.7, 212.2)	0.066^a^	0.968
Month				25.844^a^	0.258
January	86 (8.6%)	42 (10.8%)	2 (3.8%)		
February	76 (7.6%)	22 (5.6%)	4 (7.7%)		
March	114 (11.4%)	50 (12.8%)	1 (1.9%)		
April	80 (8.0%)	34 (8.7%)	5 (9.6%)		
May	85 (8.5%)	26 (6.7%)	4 (7.7%)		
June	82 (8.2%)	36 (9.2%)	4 (7.7%)		
July	87 (8.7%)	32 (8.2%)	6 (11.5%)		
August	75 (7.5%)	23 (5.9%)	8 (15.4%)		
September	70 (7.0%)	23 (5.9%)	3 (5.8%)		
October	59 (5.9%)	29 (7.4%)	3 (5.8%)		
November	103 (10.3%)	30 (7.7%)	8 (15.4%)		
December	80 (8.0%)	43 (11.0%)	4 (7.7%)		

PLSC, posterior and lateral semicircular canals (multicanal involvement); IQR, interquartile range. ^a^Kruskal-Wallis test; ^b^chi-square test.

When examining the seasonal distribution, there were no significant associations between BPPV onset and the four seasons. The percentage of cases in spring was 28.0% for multicanal, 28.2% for horizontal, and 19.2% for mixed-type. In summer, the percentages were 24.5, 23.3, and 34.6%, respectively. For autumn, they were 23.3, 21.0, and 26.9%, and for winter, 24.3, 27.4, and 19.2% (chi-square test, χ^2^ = 6.649, *p* = 0.355).

Regarding meteorological variables, no significant associations were found between BPPV onset and ambient temperature, relative humidity, barometric pressure, rainfall, or sunshine hours. The median external ambient temperature recorded on the dates of BPPV onset was 10.1 °C for PLSC, 9.85 °C for horizontal canal alone, and 13.3 °C for mixed-type (Kruskal-Wallis test, H = 3.144, *p* = 0.208). The median relative humidity was 58.9% for PLSC, 58.0% for horizontal, and 58.45% for mixed-type (Kruskal-Wallis test, H = 1.528, *p* = 0.466). The median barometric pressure was 909.1 hPa for PLSC, 908.7 hPa for horizontal, and 908.1 hPa for mixed-type (Kruskal-Wallis test, H = 1.344, *p* = 0.511). The median rainfall was 23.0 mm for PLSC, 23.6 mm for horizontal, and 23.3 mm for mixed-type (Kruskal-Wallis test, H = 0.586, *p* = 0.746). The median sunshine duration was 198.6 h for PLSC, 202.05 h for horizontal, and 204.6 h for mixed-type (Kruskal-Wallis test, H = 0.066, *p* = 0.968).

In conclusion, the study found no significant associations between BPPV onset and the local meteorological variables examined, including ambient temperature, relative humidity, barometric pressure, rainfall, or sunshine hours (Table 1).

#### 3.4.2 Multivariate analysis with BPPV onset

For the comparison “Posterior canal vs. Mixed-type,” the analysis identified atmospheric pressure, gender, and the affected side as the main influencing factors. Specifically, for each unit increase in atmospheric pressure, the odds of belonging to the posterior canal subtype increased by 5.3% (*p* = 0.016), suggesting that higher pressure may promote otolith displacement in the posterior canal. Regarding gender, the odds for male patients belonging to the posterior canal subtype were significantly reduced to 42.2% of that for females (*p* = 0.035), indicating a potential protective effect of male gender against posterior canal BPPV. Most importantly, the effect of the affected side was extremely significant: compared to bilateral involvement, unilateral involvement on either the left (OR = 63.741) or right (OR = 208.817) side vastly increased the odds of having the posterior canal subtype, with the right side being more susceptible (*p* < 0.001).

For the comparison “Horizontal canal vs. Mixed-type,” the pattern of results was similar but with subtle differences. Atmospheric pressure also showed a significant effect, with each unit increase raising the odds of the horizontal canal subtype by 5.2% (*p* = 0.020), indicating a consistent influence of this meteorological factor on both non-mixed subtypes. However, the effect of gender was attenuated; although males showed a trend towards lower odds (OR = 0.482), it did not reach statistical significance (*p* = 0.081), suggesting a less definitive role of gender in horizontal canal BPPV. The affected side remained a strong predictor, with unilateral involvement on either the left or right side significantly increasing the odds of the horizontal canal subtype (OR = 52.689 and 118.541, respectively, *p* < 0.001), although the effect sizes were slightly lower than for the posterior canal. Notably, other meteorological factors (monthly average temperature, humidity, rainfall, sunshine duration) and seasonal variation showed no significant influence in either comparison, suggesting their direct predictive value for BPPV subtyping is limited.

In summary, increased atmospheric pressure may be a common environmental risk factor for non-mixed BPPV subtypes, while gender differences appear to primarily affect posterior canal rather than horizontal canal BPPV. Most crucially, unilateral involvement (particularly on the right side) is the strongest predictor for distinguishing non-mixed from mixed-type BPPV, a finding of significant value for clinical subtyping assessment (Table 2).

**Table 2:** Multivariate analysis with BPPV onset.

Variable	Category	Posterior canal vs. mixed-type				Horizontal canal vs. mixed-type			
		B	*p* value	Exp(B)	95% CI	B	*p* value	Exp(B)	95% CI
Age	<60 years	−0.353	0.432	0.702	0.291 to 1.695	−0.345	0.449	0.708	0.289 to 1.731
	≥60 years	(Ref)				(Ref)			
Sex	Male	−0.863	**0.035**	0.422	0.189 to 0.943	−0.730	0.081	0.482	0.213 to 1.093
	Female	(Ref)				(Ref)			
Side	Left	4.155	**<0.001**	63.741	21.173 to 191.887	3.964	**<0.001**	52.689	15.087 to 184.013
	Right	5.341	**<0.001**	208.817	60.136 to 725.105	4.775	**<0.001**	118.541	29.856 to 470.662
	Bilateral	(Ref)				(Ref)			
Season	Spring	0.995	0.312	2.705	0.394 to 18.580	1.001	0.317	2.721	0.384 to 19.296
	Summer	−0.002	0.999	0.998	0.067 to 14.897	0.171	0.903	1.186	0.075 to 18.672
	Autumn	−0.216	0.781	0.806	0.176 to 3.696	−0.271	0.732	0.762	0.161 to 3.608
	Winter	(Ref)				(Ref)			
Temperature		0.002	0.966	1.002	0.899 to 1.118	−0.011	0.839	0.989	0.885 to 1.105
Humidity		0.035	0.205	1.036	0.981 to 1.093	0.029	0.301	1.029	0.974 to 1.088
Pressure		0.052	**0.016**	1.053	1.010 to 1.098	0.050	**0.020**	1.052	1.008 to 1.097
Rainfall		0.000	0.957	1.000	0.992 to 1.009	0.001	0.838	1.001	0.992 to 1.010
Sunshine		**0.010**	0.147	1.010	0.997 to 1.023	**0.010**	0.164	1.010	0.996 to 1.023

B, regression coefficient; Exp(B), odds ratio; CI, confidence interval; Ref, reference category. Statistical significance was set at *p* < 0.05. Bold values indicate statistical significance (*p* < 0.05).

#### 3.4.3 Univariate analysis with MC-BPPV onset

The study categorized MC-BPPV into two groups: “same type” (bilateral posterior/horizontal canals) with 15 cases, and “different type” (posterior-horizontal, anterior-horizontal, or posterior–anterior canals) with 37 cases. The results showed no significant difference in age between the two groups, with a mean age of 54.53 years for the same type and 52.38 years for the different type (independent samples t-test, t = 0.514, *p* = 0.610).

In terms of sex distribution, there was a significant difference. All 15 cases in the same type group were female (100.0%), while in the different type group, 14 cases were male (37.8%) and 23 cases were female (62.2%) (chi-square test, χ^2^ = 5.963, *p* = 0.015), indicating that sex is a statistically significant factor in the incidence of MC-BPPV under this classification.

Regarding seasonal distribution, there were no significant differences observed. The percentage of cases in spring was 26.7% for the same type and 16.2% for the different type. In summer, the percentages were 46.7 and 29.7%, respectively. For autumn, they were 20.0 and 29.7%, and for winter, 6.7 and 24.3% (chi-square test, χ^2^ = 3.486, *p* = 0.326).

When examining meteorological variables, temperature and sunshine duration were found to be statistically significant factors. The median temperature was 20.3 °C for the same type and 11.9 °C for the different type (Mann–Whitney U test, U = 176.0, *p* = 0.040). The mean sunshine duration was 216.13 h for the same type and 189.82 h for the different type (independent samples t-test, t = 2.196, *p* = 0.033).

Other meteorological variables such as humidity, barometric pressure, and rainfall did not show significant associations with MC-BPPV onset. The median humidity was 56% for the same type and 61.5% for the different type (Mann–Whitney U test, U = 317.5, *p* = 0.419). The median barometric pressure was 918.8 hPa for the same type and 907.5 hPa for the different type (Mann–Whitney U test, U = 227.0, *p* = 0.307). The median rainfall was 34.6 mm for the same type and 21.9 mm for the different type (Mann–Whitney U test, U = 205.5, *p* = 0.145) (Table 3).

**Table 3:** Single factor analysis with MC-BPPV onset.

Variable	Same type (*n* = 15)	Different type (*n* = 37)	*t*/*χ*^2^/*W* value	*p* value
Age	54.53 ± 16.00	52.38 ± 12.70	0.514	0.610
Sex				
Male	0 (0.0%)	14 (37.8%)	5.963	0.015*
Female	15 (100.0%)	23 (62.2%)		
Season				
Spring	4 (26.7%)	6 (16.2%)	3.486	0.326
Summer	7 (46.7%)	11 (29.7%)		
Autumn	3 (20.0%)	11 (29.7%)		
Winter	1 (6.7%)	9 (24.3%)		
Temperature	20.3 (5.6, 23.7)	11.9 (1.45, 22.2)	176.0	0.040*
Humidity	56 (48, 72)	61.5 (50.2, 78.8)	317.5	0.419
Barometric pressure	918.8 (898.6, 925.2)	907.5 (898.7, 910.8)	227.0	0.307
Rainfall	34.6 (15.8, 103.1)	21.9 (8.6, 87.25)	205.5	0.145
Sunshine duration	216.13 ± 49.15	189.82 ± 34.47	2.196	0.033*

^*^p < 0.05.

#### 3.4.4 Multivariate analysis with MC-BPPV onset

This analysis aimed to investigate the potential associations between various factors and the incidence rates of MC-BPPV, particularly when stratified by different canal involvement patterns, namely same-type (bilateral posterior/horizontal canals) and different-type (posterior-horizontal, anterior-horizontal, or posterior–anterior canals).

The analysis revealed a significant association between sunshine duration and the incidence rates of MC-BPPV (*p* = 0.039). Specifically, this relationship was observed after controlling for other covariates and was closely linked to solar irradiation parameters. The regression coefficient (B) for sunshine duration was −0.02, with an odds ratio (Exp(B)) of 0.98. The 95% confidence interval for Exp(B) ranged from 0.961 to 0.999, indicating a statistically significant effect.

In contrast, gender did not show a significant association with MC-BPPV onset in this analysis. The regression coefficient for gender was −20.891, with an Exp(B) value of 0.998 and a significance level of 0.998, suggesting no significant impact on the incidence rates of MC-BPPV (Table 4).

**Table 4:** Multivariate analysis with MC-BPPV onset.

Variable	B	Significance	Exp(B)	95% confidence interval for Exp(B)	
				Lower limit	Upper limit
Sunshine time	−0.02	0.039*	0.98	0.961	0.999
Sex	−20.891	0.998	0	0	

B, regression coefficient; Exp(B), odds ratio; CI, confidence interval. Statistical significance was set at *p* < 0.05. The exact *p*-value for sunshine duration is 0.039; *p < 0.05.

## 4 Discussion

The intricate relationship between meteorological factors, seasonal variations, and the incidence of benign paroxysmal positional vertigo (BPPV) was thoroughly explored in this study through both univariate and multivariate analyses. The findings revealed significant associations between BPPV onset and monthly variations, as well as humidity levels, after accounting for the complexity of environmental factors. Notably, mixed-type BPPV (MC-BPPV) demonstrated additional correlations with gender, temperature, and sunshine duration, suggesting potential distinctions in the pathogenesis between mixed and non-mixed subtypes that warrant further investigation.

Previous epidemiological studies have consistently identified higher BPPV prevalence during colder months, particularly in autumn and winter (2, 4, 8, 10, 17). This seasonality is often attributed to cold-induced sympathetic activation, which may alter cardiovascular parameters and blood composition, subsequently affecting microcirculation (17). This observation can be further explained by two additional factors supported by previous research. First, the higher frequency of upper respiratory tract infections during colder months has been linked to BPPV onset, potentially through inflammatory mechanisms affecting the inner ear (6, 17). Second, reduced sunlight exposure during these months leads to lower vitamin D levels, which may compromise otoconial stability and increase susceptibility to detachment (4, 9, 10). Our findings align with these seasonal patterns, showing peak incidence in March (11.28%) and the lowest rates in October (6.36%). These fluctuations might reflect combined effects of cultural factors (e.g., Chinese New Year, National Day holidays) and regional climate characteristics, necessitating larger-scale studies for confirmation (17).

Age and gender disparities in BPPV epidemiology were reaffirmed, with a 1:2.1 male-to-female ratio and the highest prevalence in the 51-70-year-old age group, consistent with prior reports (15, 16). Notably, women under 60 exhibited the highest susceptibility, while men over 60 showed the lowest incidence. Comparable prevalence rates emerged between women ≥60 and men <60. Established risk factors, including hormonal fluctuations, osteoporosis, and metabolic disorders (15), may explain these gender differences, though subgroup analyses measuring hormone and vitamin D levels are required for validation. The disappearance of gender disparity in octogenarians suggests alternative pathophysiology in this population (15).

Etiological classification revealed idiopathic BPPV predominance (50–97%), with posterior canal involvement (68.97%) and right-sided predominance matching previous anatomical observations (6, 9). Canalithiasis accounted for 91.87% of cases, compared to 4.6% cupulolithiasis, while mixed-type BPPV occurred in 3.6%, aligning with previously reported ranges of 4.6–9.3% (8, 9). Mixed-type presentations most frequently combined posterior-horizontal canal involvement (71.15%), followed by bilateral posterior canals (19.23%), with bilateral horizontal canals being the rarest (9.26%). Simultaneous bilateral onset predominated (55.77%), contrasting with left (30.77%) and right (13.46%) unilateral cases. While literature associates multi-canal involvement with trauma or idiopathic causes, emerging evidence emphasizes otologic comorbidities like labyrinthitis—a relationship requiring detailed premorbid data for verification (8, 9).

Initial univariate analysis showed no significant meteorological associations, potentially confounded by humidity’s indirect relationship with atmospheric pressure and rainfall. Multivariate modeling subsequently identified monthly variations as statistically significant, though temperature, humidity, pressure, rainfall, and sunshine duration showed no independent effects. Meta-analytic evidence (17) highlights increased cold-season incidence (January–March, December) with hemispheric variations, possibly mediated by reduced sunlight exposure impairing vitamin D synthesis (4, 10). It is important to note that the discussion on vitamin D remains speculative, as serum vitamin D levels were not measured in this study. This constitutes a limitation of our work. Our observed March peak and October trough might reflect unique regional factors, including holiday patterns and geographic climate features.

Subgroup analysis of 52 mixed BPPV cases revealed gender, temperature, and sunshine duration associations in univariate testing, with sunshine duration remaining significant in multivariate models. Temperature correlations remain controversial, with studies reporting both negative (4, 8, 10) and positive (9, 17) associations. Three mechanistic hypotheses emerge: (1) Cold-month vitamin D deficiency affecting otoconial metabolism (4, 10); (2) Seasonal viral infections/allergies exacerbating inner ear vulnerability (6, 17); (3) Vascular and weather-induced activity changes, where cold-induced vasoconstriction or pressure changes could affect labyrinthine blood flow and endolymphatic homeostasis. This pathway is supported by evidence linking vascular risk factors to other inner ear disorders, suggesting a shared susceptibility of the labyrinth to microcirculatory compromise (18). Divergent conclusions across studies may arise from methodological variations. First, single-variable climatic analyses (e.g., temperature alone) lack precision compared to multifactorial approaches. Second, temporal discrepancies between symptom onset and medical consultation may distort findings. Third, the use of monthly averaged meteorological data, as in our study, may mask short-term, acute weather fluctuations that could be more relevant triggers, representing another methodological limitation. While sunlight duration correlations remain debated (4, 7, 10, 17, 19–21), mixed BPPV’s unique association with trauma history and otologic comorbidities suggests distinct etiopathogenesis requiring targeted investigation. The proposed vitamin D synthesis pathway through sunlight exposure offers a plausible biological mechanism for these environmental associations (4, 19).

Our study underscores the complex interplay between meteorological factors and BPPV incidence. While significant associations were found between BPPV onset and monthly variations and humidity levels, further research is needed to elucidate the underlying mechanisms and potential regional differences. Future studies should employ robust multifactorial analyses and consider additional variables, such as directly measured vitamin D levels and otologic comorbidities, to provide a comprehensive understanding of the etiology and pathogenesis of BPPV. Conclusion.

## 5 Conclusion

BPPV incidence demonstrates significant associations with meteorological parameters and monthly cyclical variations, while MC-BPPV likely exhibits distinct pathogenic mechanisms compared to non-mixed subtypes. Current epidemiological evidence indicates heightened BPPV risk under conditions of diminished light exposure and elevated humidity levels, supporting clinical recommendations for increased outdoor activity and residence in drier environments as preventive measures. However, this study’s limitations regarding comorbid condition documentation and prognostic evaluations must be acknowledged. Future investigations should prioritize longitudinal analyses of subtype-specific outcomes and comorbidity impacts to elucidate potential temporal delay patterns in these associations. A comprehensive elucidation of these complex interactions remains crucial for advancing targeted therapeutic strategies and optimizing BPPV prevention protocols.

## Data Availability

The original contributions presented in the study are included in the article/supplementary material, further inquiries can be directed to the corresponding author.
